# Supplementary material for: Analysis of Whole-Genome Sequences of Pathogenic Gram-Positive and Gram-Negative Isolates from the Same Hospital Environment to Investigate Common Evolutionary Trends Associated with Horizontal Gene Exchange, Mutations and DNA Methylation Patterning
Source: Microorganisms. 2023 Jan 27;11(2):323. doi: 10.3390/microorganisms11020323 (PMC9961978; doi:10.3390/microorganisms11020323)
Supplement: Supplementary file 1 [file microorganisms-11-00323-s001.zip › Supplementary Table S1.pdf]

Supplementary Table S1. Antibiotic resistance genes found in genomes of the isolates

Strain	Gene	Mechanism of action	In GI	Resistance
Klebsiella pneumonia **13/97**				
13/97: Chr	*eptB* [218462..220135]	target alteration		peptide
	arnT [300397..302052]	target alteration		peptide
	CRP [411005..411637]	efflux		macrolide, fluoroquinolone, penam
	rsmA [1096931..1097116]	efflux		fluoroquinolone, diaminopyrimidine, phenicol
	kpnH [1105114..1106649]	efflux		macrolide, fluoroquinolone, aminoglycoside, carbapenem, cephalosporin, penam, peptide, penem
	kpnG [1106665..1107837]	efflux		macrolide, fluoroquinolone, aminoglycoside, carbapenem, cephalosporin, penam, peptide, penem
	emrR [1107963..1108493]	efflux		fluoroquinolone
	adeF [1175284..1178436]	efflux	GI#1	fluoroquinolone, tetracycline
	oqxA [1178460..1179635]	efflux	GI#1	fluoroquinolone, glycylcycline, tetracycline, diaminopyrimidine, nitrofuran
	adeF [1415019..1418132]	efflux		fluoroquinolone, tetracycline
	adeF [1422608..1425721]	efflux		fluoroquinolone, tetracycline
	adeF	efflux	GI#4	fluoroquinolone,

	[1463467..1466580]			tetracycline
	baeR [1769838..1770560]	efflux		aminoglycoside, aminocoumarin
	H-NS [2278652..2279059]	efflux		macrolide, fluoroquinolone, cephalosporin, cephamycin, penam, tetracycline
	marA [2865787..2866161]	efflux; reduced permeability to antibiotic		fluoroquinolone, monobactam, carbapenem, cephalosporin, glycylcycline, cephamycin, penam, tetracycline, rifamycin, phenicol, triclosan, penem
	SHV-11 [2886264..2887124]	beta-lactamase		carbapenem, cephalosporin, penam
	kpnF [2927174..2927503]	efflux		macrolide, aminoglycoside, cephalosporin, tetracycline, peptide, rifamycin
	kpnE [2927490..2927852]	efflux		macrolide, aminoglycoside, cephalosporin, tetracycline, peptide, rifamycin
	ompK37 [3052873..3053997]	reduced permeability to antibiotic		monobactam, carbapenem, cephalosporin, cephamycin, penam, penem
	ompA [3547574..3548644]	reduced permeability to antibiotic		monobactam, carbapenem, cephalosporin, cephamycin, penam, penem
	msbA [3598015..3599763]	efflux		nitroimidazole
	ampH	beta-lactamase		cephalosporin,

	[4317245..4318405]			penam
	LptD [4619756..4622104]	efflux		carbapenem, peptide, aminocoumarin, rifamycin
	FosA6 [4762321..4762740]	inactivation		fosfomycin
	UhpT E350Q [39078..40469]	mutation		fosfomycin
	EF-Tu R234F [426683..427867]	mutation		elfamycin
	Pbp3 D350N, S357N [4572290..4574056]	mutation		cephalosporin, cephamycin, penam
	MarR G103S, Y137H [2865332..2865766]	mutation		fluoroquinolone, cephalosporin, glycylcycline, penam, tetracycline, rifamycin, phenicol, triclosan
Pseudomonas aeruginosa 16/222				
16/222: Chr	*triA* [185842..186945]	efflux		triclosan
	triB [186942..188012]	efflux		triclosan
	triC [188009..191056]	efflux		triclosan
	mexA [478590..479741]	efflux		macrolide, fluoroquinolone, monobactam, carbapenem, cephalosporin, cephamycin, penam, tetracycline, peptide, aminocoumarin, diaminopyrimidine, sulfonamide, phenicol, penem
	mexB [479757..482897]	efflux		macrolide, fluoroquinolone, monobactam, carbapenem, cephalosporin,

				cephamycin, penam, tetracycline, peptide, aminocoumarin, diaminopyrimidine, sulfonamide, phenicol, penem
	oprM [482899..484356]	efflux		macrolide, fluoroquinolone, monobactam, aminoglycoside, carbapenem, cephalosporin, cephamycin, penam, tetracycline, peptide, acridine dye, aminocoumarin, diaminopyrimidine, sulfonamide, phenicol, penem, disinfecting agents and intercalating dyes
	opmD [805899..807362]	efflux		fluoroquinolone, tetracycline, acridine dye, disinfecting agents and intercalating dyes
	mexI [807359..810448]	efflux		fluoroquinolone, tetracycline, acridine dye, disinfecting agents and intercalating dyes
	mexH [810461..811573]	efflux		fluoroquinolone, tetracycline, acridine dye, disinfecting agents and intercalating dyes
	mexG [811581..812027]	efflux		fluoroquinolone, tetracycline, acridine dye, disinfecting agents and intercalating dyes
	bcr-1 [956045..957253]	efflux		bicyclomycin
	APH(3')-IIb	inactivation		aminoglycoside

	[973952..974758]			
	PDC-19a [987004..988197]	inactivation		monobactam, carbapenem, cephalosporin
	yajC [1324844..1325182]	efflux		fluoroquinolone, cephalosporin, glycylcycline, penam, tetracycline, rifamycin, phenicol, triclosan
	mexL [1483064..1483702]	efflux		macrolide, tetracycline, triclosan
	mexJ [1483798..1484901]	efflux		macrolide, tetracycline, triclosan
	mexK [1484906..1487983]	efflux		macrolide, tetracycline, triclosan
	arnA [1603989..1605977]	target alteration	GI#6	peptide
	mexP [1644197..1645354]	efflux		macrolide, carbapenem, tetracycline, acridine dye, diaminopyrimidine, phenicol, disinfecting agents and intercalating dyes
	mexQ [1645351..1648512]	efflux		macrolide, carbapenem, tetracycline, acridine dye, diaminopyrimidine, phenicol, disinfecting agents and intercalating dyes
	opmE [1648509..1649984]	efflux		macrolide, carbapenem, tetracycline, acridine dye, diaminopyrimidine, phenicol, disinfecting agents and intercalating dyes
	cpxR	efflux		macrolide,

	[2000915..2001592]			fluoroquinolone, monobactam, aminoglycoside, carbapenem, cephalosporin, cephamycin, penam, tetracycline, peptide, aminocoumarin, diaminopyrimidine, sulfonamide, phenicol, penem
	cprS [2133964..2135259]	target alteration; efflux		peptide
	cprR [2135256..2135927]	target alteration; efflux		peptide
	muxA [2942578..2943858]	efflux		macrolide, monobactam, tetracycline, aminocoumarin
	muxB [2943855..2946986]	efflux		macrolide, monobactam, tetracycline, aminocoumarin
	muxC [2946983..2950093]	efflux		macrolide, monobactam, tetracycline, aminocoumarin
	opmB [2950090..2951586]	efflux		macrolide, monobactam, tetracycline, aminocoumarin
	oprN [2983262..2984680]	efflux		fluoroquinolone, diaminopyrimidine, phenicol
	mexF [2984677..2987865]	efflux		fluoroquinolone, diaminopyrimidine, phenicol
	mexE [2987887..2989131]	efflux		fluoroquinolone, diaminopyrimidine, phenicol
	SoxR G74R [3288231..3288701]	mutation		fluoroquinolone, cephalosporin, glycylcycline, penam,

				tetracycline, acridine dye, rifamycin, phenicol, triclosan, disinfecting agents and intercalating dyes
	adeF [3688321..3691458]	efflux		fluoroquinolone, tetracycline
	parR [3982452..3983159]	efflux; reduced permeability to antibiotic		macrolide, fluoroquinolone, monobactam, aminoglycoside, carbapenem, cephalosporin, cephamycin, penam, tetracycline, acridine dye, phenicol, penem, disinfecting agents and intercalating dyes
	parS [3983160..3984446]	efflux; reduced permeability to antibiotic		macrolide, fluoroquinolone, monobactam, aminoglycoside, carbapenem, cephalosporin, cephamycin, penam, tetracycline, acridine dye, phenicol, penem, disinfecting agents and intercalating dyes
	mexN [4483260..4486370]	efflux		phenicol
	mexM [4486367..4487524]	efflux		phenicol
	pmpM [4579586..4581019]	efflux		fluoroquinolone, aminoglycoside, benzalkonium chloride
	fosA [4846590..4846997]	inactivation		fosfomycin
	rsmA [5075506..5075691]	efflux		fluoroquinolone, diaminopyrimidine, phenicol
	catB7	inactivation	GI#22	phenicol

	[5355382..5356020]			
	mexV [5522727..5523857]	efflux		macrolide, fluoroquinolone, tetracycline, acridine dye, phenicol, disinfecting agents and intercalating dyes
	mexW [5523908..5526964]	efflux		macrolide, fluoroquinolone, tetracycline, acridine dye, phenicol, disinfecting agents and intercalating dyes
	oprJ [5992953..5994392]	efflux		macrolide, fluoroquinolone, aminoglycoside, cephalosporin, penam, tetracycline, aminocoumarin, diaminopyrimidine, phenicol
	mexD [5994398..5997529]	efflux		macrolide, fluoroquinolone, aminoglycoside, cephalosporin, penam, tetracycline, aminocoumarin, diaminopyrimidine, phenicol
	mexC [5997557..5998621]	efflux		macrolide, fluoroquinolone, aminoglycoside, cephalosporin, penam, tetracycline, aminocoumarin, diaminopyrimidine, phenicol
	basS [6221749..6223182]	target alteration; efflux		peptide
	opmH [6441985..6443433]	efflux		triclosan
	emrE [6463987..6464319]	efflux		aminoglycoside

	OXA-488 [7066768..7067556]	beta-lactamase		carbapenem, cephalosporin, penam
	gyrA T83I [2036983..2039754]	mutation	GI#7	fluoroquinolone
	mexR [477872..478315]	target alteration; efflux		macrolide, fluoroquinolone, monobactam, carbapenem, cephalosporin, cephamycin, penam, tetracycline, peptide, aminocoumarin, diaminopyrimidine, sulfonamide, phenicol, penem
	NalC S209R, G71E [1437027..1437668]	mutation		macrolide, fluoroquinolone, monobactam, carbapenem, cephalosporin, cephamycin, penam, tetracycline, peptide, aminocoumarin, diaminopyrimidine, sulfonamide, phenicol, penem
	mexT [2989362..2990276]	efflux		fluoroquinolone, diaminopyrimidine, phenicol
	MexS V73A [2990497..2991516]	mutation		fluoroquinolone, diaminopyrimidine, phenicol
	mexZ [3686021..3686950]	efflux		macrolide, fluoroquinolone, aminoglycoside, carbapenem, cephalosporin, cephamycin, penam, tetracycline, acridine dye, phenicol, disinfecting agents and intercalating dyes
	nfxB [5998881..5999444]	efflux		macrolide, fluoroquinolone,

				cephalosporin, penam, tetracycline, aminocoumarin, diaminopyrimidine, phenicol
Escherichia coli 19/278				
19/278: Chr	*gadX* [239242..240066]	efflux		macrolide, fluoroquinolone, penam
	gadW [240435..241163]	efflux		macrolide, fluoroquinolone, penam
	mdtF [241526..244639]	efflux		macrolide, fluoroquinolone, penam
	mdtE [244664..245821]	efflux		macrolide, fluoroquinolone, penam
	CRP [419421..420053]	efflux		macrolide, fluoroquinolone, penam
	acrF [487895..490999]	efflux		fluoroquinolone, cephalosporin, cephamycin, penam
	acrE [491011..492168]	efflux		fluoroquinolone, cephalosporin, cephamycin, penam
	acrS [492567..493229]	efflux		fluoroquinolone, cephalosporin, glycylcycline, cephamycin, penam, tetracycline, rifamycin, phenicol, triclosan
	bacA [703118..703939]	target alteration		peptide
	tolC [726317..727798]	efflux		macrolide, fluoroquinolone, aminoglycoside, carbapenem, cephalosporin, glycylcycline, cephamycin, penam,

				tetracycline, peptide, aminocoumarin, rifamycin, phenicol, triclosan, penem
	rsmA [1195009..1195194]	efflux		fluoroquinolone, diaminopyrimidine, phenicol
	emrB [1200444..1201982]	efflux		fluoroquinolone
	emrA [1201999..1203171]	efflux		fluoroquinolone
	emrR [1203298..1203828]	efflux		fluoroquinolone
	acrD [1399055..1402168]	efflux		aminoglycoside
	evgS [1490405..1493998]	efflux	GI#6	macrolide, fluoroquinolone, penam, tetracycline
	evgA [1494003..1494617]	efflux	GI#6	macrolide, fluoroquinolone, penam, tetracycline
	emrK [1495033..1496196]	efflux	GI#6	tetracycline
	emrY [1496196..1497734]	efflux	GI#6	tetracycline
	pmrF [1700855..1701823]	target alteration		peptide
	yojI [1760355..1761998]	efflux		peptide
	baeR [1897456..1898178]	efflux		aminoglycoside, aminocoumarin
	baeS [1898175..1899578]	efflux		aminoglycoside, aminocoumarin
	mdtC [1900991..1904068]	efflux		aminocoumarin
	mdtB [1904069..1907191]	efflux		aminocoumarin
	mdtA [1907191..1908438]	efflux		aminocoumarin
	ugd [1962819..1963985]	target alteration	GI#9	peptide

	emrE [2191298..2191630]	efflux		macrolide
	kpnE [2535262..2535627]	efflux		macrolide, aminoglycoside, cephalosporin, tetracycline, peptide, rifamycin
	kpnF [2535614..2535943]	efflux		macrolide, aminoglycoside, cephalosporin, tetracycline, peptide, rifamycin
	marA [2574143..2574526]	efflux; reduced permeability to antibiotic		fluoroquinolone, monobactam, carbapenem, cephalosporin, glycylcycline, cephamycin, penam, tetracycline, rifamycin, phenicol, triclosan, penem
	H-NS [2876067..2876480]	efflux		macrolide, fluoroquinolone, cephalosporin, cephamycin, penam, tetracycline
	mdtH [3102600..3103808]	efflux		fluoroquinolone
	mdtG [3112439..3113665]	efflux		fosfomycin
	msbA [3238549..3240297]	efflux		nitroimidazole
	mdfA [3350187..3351419]	efflux		tetracycline, benzalkonium chloride, rhodamine
	kdpE [3494059..3494736]	efflux		aminoglycoside
	acrA [3708910..3710103]	efflux		fluoroquinolone, cephalosporin, glycylcycline, penam, tetracycline, rifamycin, phenicol, triclosan

	acrB [3710126..3713275]	efflux		fluoroquinolone, cephalosporin, glycylcycline, penam, tetracycline, rifamycin, phenicol, triclosan
	ampH [3802035..3803192]	beta-lactamase		cephalosporin, penam
	ampC [4527354..4528520]	beta-lactamase		cephalosporin, penam
	eptA [4628729..4630372]	target alteration		peptide
	mdtN [4659599..4660630]	efflux		nucleoside, acridine dye, disinfecting agents and intercalating dyes
	mdtO [4660630..4662681]	efflux		nucleoside, acridine dye, disinfecting agents and intercalating dyes
	mdtP [4662678..4664144]	efflux		nucleoside, acridine dye, disinfecting agents and intercalating dyes
	cpxA [4922364..4923737]	efflux		aminoglycoside, aminocoumarin
19/278: pl#1	*dfrA17* [39937..40410]	target replacement		diaminopyrimidine
	tet(B) [41983..43188]	efflux		tetracycline
	sul2 [48365..49180]	target replacement		sulfonamide
	APH(3'')-Ib [49241..50044]	inactivation		aminoglycoside
	APH(6)-Id [50044..50880]	inactivation		aminoglycoside
	mphA [58802..59707]	inactivation		macrolide
	TEM-1 [61118..61978]	beta-lactamase		monobactam, cephalosporin, penam, penem
19/278: pl#2	*CTX-M-55* [46844..47719]	inactivation		cephalosporin

19/278: Chr	UhpT E350Q [32210..33601]	mutation		fosfomycin
	EF-Tu R234F [434843..436027]	mutation		elfamycin
	ParC S80I [739940..742198]	mutation		fluoroquinolone
	GyrA D87N, S83L [1729526..1732153]	mutation		fluoroquinolone
	Pbp3 D350N, S357N [4146513..4148279]	mutation		cephalosporin, cephamycin, penam
	MarR Y137H, G103S, S3N [2574547..2574981]	mutation		fluoroquinolone, cephalosporin, glycylcycline, penam, tetracycline, rifamycin, phenicol, triclosan
	SoxR G74R [4692022..4692486]	mutation		fluoroquinolone, cephalosporin, glycylcycline, penam, tetracycline, rifamycin, phenicol, triclosan
19/278: pl#1	*tetR* [41278..41901]	target alteration; efflux		tetracycline
Klebsiella pneumonia 20/245				
20/245: Chr	*eptB* [223563..225236]	target alteration		peptide
	arnT [285276..286931]	target alteration		peptide
	CRP [399702..400334]	efflux		macrolide, fluoroquinolone, penam
	crcB [835924..836337]	efflux		aminoglycoside
	rsmA [1127560..1127745]	efflux		fluoroquinolone, diaminopyrimidine, phenicol
	kpnH [1135473..1137011]	efflux		macrolide, fluoroquinolone, aminoglycoside, carbapenem,

				cephalosporin, penam, peptide, penem
	kpnG [1137027..1138199]	efflux		macrolide, fluoroquinolone, aminoglycoside, carbapenem, cephalosporin, penam, peptide, penem
	emrR [1138325..1138855]	efflux		fluoroquinolone
	adeF [1183126..1186278]	efflux		fluoroquinolone, tetracycline
	oqxA [1186302..1187477]	efflux		fluoroquinolone, glycylcycline, tetracycline, diaminopyrimidine, nitrofuran
	adeF [1364355..1367468]	efflux		fluoroquinolone, tetracycline
	baeR [1665755..1666477]	efflux		aminoglycoside, aminocoumarin
	H-NS [2181193..2181600]	efflux		macrolide, fluoroquinolone, cephalosporin, cephamycin, penam, tetracycline
	marA [2735216..2735590]	efflux; reduced permeability to antibiotic		fluoroquinolone, monobactam, carbapenem, cephalosporin, glycylcycline, cephamycin, penam, tetracycline, rifamycin, phenicol, triclosan, penem
	SHV-207 [2755694..2756554]	beta-lactamase		carbapenem, cephalosporin, penam
	kpnF [2795737..2796066]	efflux		macrolide, aminoglycoside, cephalosporin,

				tetracycline, peptide, rifamycin
	kpnE [2796053..2796415]	efflux		macrolide, aminoglycoside, cephalosporin, tetracycline, peptide, rifamycin
	ompK37 [2984505..2985629]	reduced permeability to antibiotic		monobactam, carbapenem, cephalosporin, cephamycin, penam, penem
	ompA [3422707..3423777]	reduced permeability to antibiotic		monobactam, carbapenem, cephalosporin, cephamycin, penam, penem
	msbA [3473150..3474898]	efflux		nitroimidazole
	ampH [4160179..4161339]	beta-lactamase		cephalosporin, penam
	lptD [4471754..4474069]	efflux		carbapenem, peptide, aminocoumarin, rifamycin
	fosA6 [4636455..4636874]	inactivation		fosfomycin
	UhpT E350Q [39019..40410]	mutation		fosfomycin
	EF-Tu R234F [415381..416565]	mutation		elfamycin
	Pbp3 D350N, S357N [4424261..4426027]	mutation		cephalosporin, cephamycin, penam
	MarR G103S, Y137H [2734761..2735195]	mutation		fluoroquinolone, cephalosporin, glycylcycline, penam, tetracycline, rifamycin, phenicol, triclosan
Escherichia coli 3/145				
3/145: Chr	*gadX* [242678..243502]	efflux		macrolide, fluoroquinolone,

				penam
	mdtF [244962..248075]	efflux		macrolide, fluoroquinolone, penam
	mdtE [248100..249257]	efflux		macrolide, fluoroquinolone, penam
	CRP [449651..450283]	efflux		macrolide, fluoroquinolone, penam
	acrF [503002..506106]	efflux		fluoroquinolone, cephalosporin, cephamycin, penam
	acrE [506118..507275]	efflux		fluoroquinolone, cephalosporin, cephamycin, penam
	acrS [507674..508336]	efflux		fluoroquinolone, cephalosporin, glycylcycline, cephamycin, penam, tetracycline, rifamycin, phenicol, triclosan
	bacA [707873..708694]	target alteration		peptide
	tolC [729415..730896]	efflux		macrolide, fluoroquinolone, aminoglycoside, carbapenem, cephalosporin, glycylcycline, cephamycin, penam, tetracycline, peptide, aminocoumarin, rifamycin, phenicol, triclosan, penem
	rsmA [1224393..1224578]	efflux		fluoroquinolone, diaminopyrimidine, phenicol
	emrB [1229519..1231057]	efflux		fluoroquinolone
	emrA [1231074..1232246]	efflux		fluoroquinolone

	emrR [1232373..1232903]	efflux		fluoroquinolone
	acrD [1469111..1472224]	efflux		aminoglycoside
	ampC1 [1503748..1505049]	beta-lactamase		cephalosporin, penam
	evgA [1562196..1562810]	efflux	GI#8	macrolide, fluoroquinolone, penam, tetracycline
	emrK [1563226..1564389]	efflux	GI#8	tetracycline
	emrY [1564389..1565927]	efflux	GI#8	tetracycline
	pmrF [1667680..1668648]	target alteration		peptide
	yojI [1730064..1731707]	efflux		peptide
	baeR [1875475..1876197]	efflux		aminoglycoside, aminocoumarin
	baeS [1876194..1877597]	efflux		aminoglycoside, aminocoumarin
	mdtC [1879010..1882087]	efflux		aminocoumarin
	mdtB [1882088..1885210]	efflux		aminocoumarin
	mdtA [1885210..1886577]	efflux		aminocoumarin
	ugd [1938232..1939398]	target alteration	GI#9	peptide
	kpnE [2424325..2424690]	efflux		macrolide, aminoglycoside, cephalosporin, tetracycline, peptide, rifamycin
	kpnF [2424677..2425006]	efflux		macrolide, aminoglycoside, cephalosporin, tetracycline, peptide, rifamycin
	marA [2502114..2502497]	efflux; reduced permeability to antibiotic		fluoroquinolone, monobactam,

				carbapenem, cephalosporin, glycylcycline, cephamycin, penam, tetracycline, rifamycin, phenicol, triclosan, penem
	H-NS [2785831..2786244]	efflux		macrolide, fluoroquinolone, cephalosporin, cephamycin, penam, tetracycline
	mdtH [3043754..3044962]	efflux		fluoroquinolone
	mdtG [3053593..3054819]	efflux		fosfomycin
	msbA [3179041..3180789]	efflux		nitroimidazole
	mdfA [3267358..3268590]	efflux		tetracycline, benzalkonium chloride, rhodamine
	kdpE [3474915..3475592]	efflux		aminoglycoside
	acrA [3761075..3762268]	efflux		fluoroquinolone, cephalosporin, glycylcycline, penam, tetracycline, rifamycin, phenicol, triclosan
	acrB [3762291..3765440]	efflux		fluoroquinolone, cephalosporin, glycylcycline, penam, tetracycline, rifamycin, phenicol, triclosan
	ampH [3853936..3855093]	beta-lactamase		cephalosporin, penam
	mdtM [4404016..4405248]	efflux		fluoroquinolone, lincosamide, nucleoside, acridine dye, phenicol, disinfecting agents and intercalating

				dyes
	ampC [4633798..4634964]	beta-lactamase		cephalosporin, penam
	eptA [4676873..4678516]	target alteration		peptide
	mdtN [4701060..4702091]	efflux	GI#22	nucleoside, acridine dye, disinfecting agents and intercalating dyes
	mdtO [4702091..4704142]	efflux	GI#22	nucleoside, acridine dye, disinfecting agents and intercalating dyes
	mdtP [4704139..4705605]	efflux	GI#22	nucleoside, acridine dye, disinfecting agents and intercalating dyes
3/145: pl#1	*aadA5* [57111..57899]	inactivation		aminoglycoside
	qacEdelta1 [58105..58452]	efflux		acridine dye, disinfecting agents and intercalating dyes
	sul1 [58446..59285]	target replacement		sulfonamide
	TEM-1 [61934..62794]	beta-lactamase		monobactam, cephalosporin, penam, penem
	catI [65066..65725]	inactivation		phenicol
	mphA [70478..71383]	inactivation		macrolide
	tet(B) [73614..74819]	efflux		tetracycline
3/145: Chr	EF-Tu R234F [465074..466258]	mutation		elfamycin
	GlpT E448K [1682816..1684174]	mutation		fosfomycin
	Pbp3 D350N, S357N [4213379..4215145]	mutation		cephalosporin, cephamycin, penam
	CyaA S352T [5022063..5024609]	mutation		fosfomycin

	MarR Y137H, G103S [2502517..2502951]	mutation		fluoroquinolone, cephalosporin, glycylcycline, penam, tetracycline, rifamycin, phenicol, triclosan
	SoxR G74R [4727348..4727812]	target alteration; efflux		fluoroquinolone, cephalosporin, glycylcycline, penam, tetracycline, rifamycin, phenicol, triclosan
3/145: pl#1	*tetR* [72909..73532]	target alteration; efflux		tetracycline
Staphylococcus epidermidis 597/2				
597/2.chr	*mecA* [64264..66270]	target replacement	GI#1	penam
	norA [1736479..1737642]	efflux		fluoroquinolone, acridine dye, disinfecting agents and intercalating dyes
597/2: pl#2	*mphC* [4651..5550]	inactivation		macrolide
	msrA [5649..7115]	target protection		macrolide, lincosamide, streptogramin, tetracycline, oxazolidinone, phenicol, pleuromutilin
597/2: pl#3	*blaZ* [9180..10025]	beta-lactamase		penam
Staphylococcus aureus 597/2/2				
597/2/2: Chr	*mepR* [336896..337315]	efflux		glycylcycline, tetracycline
	mgrA [697821..698264]	efflux		fluoroquinolone, cephalosporin, penam, tetracycline, peptide, acridine dye, disinfecting agents and intercalating

				dyes
	norA [705915..707081]	efflux		fluoroquinolone
	arlR [1384420..1385079]	efflux		fluoroquinolone, acridine dye, disinfecting agents and intercalating dyes
	lmrS [2172134..2173576]	efflux		macrolide, aminoglycoside, oxazolidinone, diaminopyrimidine, phenicol
	GlpT A100V [339403..340761]	mutation		fosfomycin
	MurA E291D, T396N [2094696..2095961]	mutation		fosfomycin
Staphylococcus aureus 598				
598: Chr	*mepR* [316853..317272]	efflux		glycylcycline, tetracycline
	mgrA [723742..724185]	efflux		fluoroquinolone, cephalosporin, penam, tetracycline, peptide, acridine dye, disinfecting agents and intercalating dyes
	norA [731838..733004]	efflux		fluoroquinolone
	arlR [1439202..1439861]	efflux		fluoroquinolone, acridine dye, disinfecting agents and intercalating dyes
	blaZ [1812377..1813222]	beta-lactamase		penam
	fosB [2473348..2473767]	inactivation		fosfomycin
	GlpT A100V, V213I [319364..320722]	mutation		fosfomycin
	MurA D278E,	mutation		fosfomycin

	E291D [2242253..2243518]			
Pseudomonas aeruginosa 7/157				
7/157: Chr	*triA* [185842..186945]	efflux		triclosan
	triB [186942..188012]	efflux		triclosan
	triC [188009..191056]	efflux		triclosan
	mexA [478587..479738]	efflux		macrolide, fluoroquinolone, monobactam, carbapenem, cephalosporin, cephamycin, penam, tetracycline, peptide, aminocoumarin, diaminopyrimidine, sulfonamide, phenicol, penem
	mexB [479754..482894]	efflux		macrolide, fluoroquinolone, monobactam, carbapenem, cephalosporin, cephamycin, penam, tetracycline, peptide, aminocoumarin, diaminopyrimidine, sulfonamide, phenicol, penem
	oprM [482896..484353]	efflux		macrolide, fluoroquinolone, monobactam, aminoglycoside, carbapenem, cephalosporin, cephamycin, penam, tetracycline, peptide, acridine dye, aminocoumarin, diaminopyrimidine, sulfonamide, phenicol, penem,

				disinfecting agents and intercalating dyes
	opmD [805895..807358]	efflux		fluoroquinolone, tetracycline, acridine dye, disinfecting agents and intercalating dyes
	mexI [807355..810444]	efflux		fluoroquinolone, tetracycline, acridine dye, disinfecting agents and intercalating dyes
	mexH [810457..811569]	efflux		fluoroquinolone, tetracycline, acridine dye, disinfecting agents and intercalating dyes
	mexG [811577..812023]	efflux		fluoroquinolone, tetracycline, acridine dye, disinfecting agents and intercalating dyes
	bcr-1 [929025..930233]	efflux		bicyclomycin
	APH(3')-IIb [946932..947738]	inactivation		aminoglycoside
	PDC-19a [959984..961177]	inactivation		monobactam, carbapenem, cephalosporin
	yajC [1297822..1298160]	efflux		fluoroquinolone, cephalosporin, glycylcycline, penam, tetracycline, rifamycin, phenicol, triclosan
	mexL [1456041..1456679]	efflux		macrolide, tetracycline, triclosan
	mexJ [1456775..1457878]	efflux		macrolide, tetracycline, triclosan
	mexK [1457883..1460960]	efflux		macrolide, tetracycline, triclosan
	arnA	target alteration		peptide

	[1599360..1601348]			
	mexP [1639568..1640725]	efflux		macrolide, carbapenem, tetracycline, acridine dye, diaminopyrimidine, phenicol, disinfecting agents and intercalating dyes
	mexQ [1640722..1643883]	efflux		macrolide, carbapenem, tetracycline, acridine dye, diaminopyrimidine, phenicol, disinfecting agents and intercalating dyes
	opmE [1643880..1645355]	efflux		macrolide, carbapenem, tetracycline, acridine dye, diaminopyrimidine, phenicol, disinfecting agents and intercalating dyes
	cpxR [1996279..1996956]	efflux		macrolide, fluoroquinolone, monobactam, aminoglycoside, carbapenem, cephalosporin, cephamycin, penam, tetracycline, peptide, aminocoumarin, diaminopyrimidine, sulfonamide, phenicol, penem
	cprS [2130567..2131862]	target alteration; efflux		peptide
	cprR [2131859..2132530]	target alteration; efflux		peptide
	muxA [2912582..2913862]	efflux		macrolide, monobactam, tetracycline,

				aminocoumarin
	muxB [2913859..2916990]	efflux		macrolide, monobactam, tetracycline, aminocoumarin
	muxC [2916987..2920097]	efflux		macrolide, monobactam, tetracycline, aminocoumarin
	opmB [2920094..2921590]	efflux		macrolide, monobactam, tetracycline, aminocoumarin
	oprN [2953264..2954682]	efflux		fluoroquinolone, diaminopyrimidine, phenicol
	adeF [2954679..2957630]	efflux		fluoroquinolone, tetracycline
	mexE [2957888..2959132]	efflux		fluoroquinolone, diaminopyrimidine, phenicol
	soxR [3256411..3256881]	target alteration; efflux		fluoroquinolone, cephalosporin, glycylcycline, penam, tetracycline, acridine dye, rifamycin, phenicol, triclosan, disinfecting agents and intercalating dyes
	adeF [3670367..3673504]	efflux		fluoroquinolone, tetracycline
	parR [3964487..3965194]	efflux; reduced permeability to antibiotic		macrolide, fluoroquinolone, monobactam, aminoglycoside, carbapenem, cephalosporin, cephamycin, penam, tetracycline, acridine dye, phenicol, penem, disinfecting agents and

				intercalating dyes
	parS [3965195..3966481]	efflux; reduced permeability to antibiotic		macrolide, fluoroquinolone, monobactam, aminoglycoside, carbapenem, cephalosporin, cephamycin, penam, tetracycline, acridine dye, phenicol, penem, disinfecting agents and intercalating dyes
	mexN [4456361..4459471]	efflux		phenicol
	mexM [4459468..4460625]	efflux		phenicol
	pmpM [4552685..4554118]	efflux		fluoroquinolone, aminoglycoside, benzalkonium chloride
	fosA [4819688..4820095]	inactivation		fosfomycin
	rsmA [5131306..5131491]	efflux		fluoroquinolone, diaminopyrimidine, phenicol
	catB7 [5407303..5407941]	inactivation	GI#24	phenicol
	mexV [5574647..5575777]	efflux		macrolide, fluoroquinolone, tetracycline, acridine dye, phenicol, disinfecting agents and intercalating dyes
	mexW [5575828..5578884]	efflux		macrolide, fluoroquinolone, tetracycline, acridine dye, phenicol, disinfecting agents and intercalating dyes
	crpP	inactivation	GI#26	fluoroquinolone,

	[5774831..5775025]			ciprofloxacin
	oprJ [6041367..6042806]	efflux		macrolide, fluoroquinolone, aminoglycoside, cephalosporin, penam, tetracycline, aminocoumarin, diaminopyrimidine, phenicol
	mexD [6042812..6045943]	efflux		macrolide, fluoroquinolone, aminoglycoside, cephalosporin, penam, tetracycline, aminocoumarin, diaminopyrimidine, phenicol
	mexC [6045971..6047035]	efflux		macrolide, fluoroquinolone, aminoglycoside, cephalosporin, penam, tetracycline, aminocoumarin, diaminopyrimidine, phenicol
	basS [6270164..6271597]	target alteration; efflux		peptide
	opmH [6490400..6491848]	efflux		triclosan
	emrE [6512401..6512733]	efflux		aminoglycoside
	OXA-488 [7116059..7116847]	beta-lactamase		carbapenem, cephalosporin, penam
	GyrA T83I [2032347..2035118]	mutation		fluoroquinolone
	mexR [477869..478312]	target alteration; efflux		macrolide, fluoroquinolone, monobactam, carbapenem, cephalosporin, cephamycin, penam, tetracycline, peptide,

				aminocoumarin, diaminopyrimidine, sulfonamide, phenicol, penem
	NalC S209R, G71E [1410005..1410646]	mutation		macrolide, fluoroquinolone, monobactam, carbapenem, cephalosporin, cephamycin, penam, tetracycline, peptide, aminocoumarin, diaminopyrimidine, sulfonamide, phenicol, penem
	mexT [2959363..2960277]	efflux		fluoroquinolone, diaminopyrimidine, phenicol
	MexS V73A [2960498..2961517]	mutation		fluoroquinolone, diaminopyrimidine, phenicol
	mexZ [3668067..3668996]	efflux		macrolide, fluoroquinolone, aminoglycoside, carbapenem, cephalosporin, cephamycin, penam, tetracycline, acridine dye, phenicol, disinfecting agents and intercalating dyes
	nfxB [6047295..6047858]	efflux		macrolide, fluoroquinolone, cephalosporin, penam, tetracycline, aminocoumarin, diaminopyrimidine, phenicol
Pseudomonas aeruginosa 91/195				
91/195: Chr	*triA* [175604..176707]	efflux		triclosan
	triB	efflux		triclosan

	[176704..177774]			
	triC [177771..180818]	efflux		triclosan
	mexA [496702..497853]	efflux		macrolide, fluoroquinolone, monobactam, carbapenem, cephalosporin, cephamycin, penam, tetracycline, peptide, aminocoumarin, diaminopyrimidine, sulfonamide, phenicol, penem
	mexB [497869..501009]	efflux		macrolide, fluoroquinolone, monobactam, carbapenem, cephalosporin, cephamycin, penam, tetracycline, peptide, aminocoumarin, diaminopyrimidine, sulfonamide, phenicol, penem
	oprM [501011..502468]	efflux		macrolide, fluoroquinolone, monobactam, aminoglycoside, carbapenem, cephalosporin, cephamycin, penam, tetracycline, peptide, acridine dye, aminocoumarin, diaminopyrimidine, sulfonamide, phenicol, penem, disinfecting agents and intercalating dyes
	opmD [856904..858367]	efflux		fluoroquinolone, tetracycline, acridine dye, disinfecting agents and

				intercalating dyes
	mexI [858364..861453]	efflux		fluoroquinolone, tetracycline, acridine dye, disinfecting agents and intercalating dyes
	mexH [861466..862578]	efflux		fluoroquinolone, tetracycline, acridine dye, disinfecting agents and intercalating dyes
	mexG [862586..863032]	efflux		fluoroquinolone, tetracycline, acridine dye, disinfecting agents and intercalating dyes
	bcr-1 [942933..944141]	efflux		bicyclomycin
	APH(3')-IIb [960851..961657]	inactivation		aminoglycoside
	PDC-1 [974013..975206]	inactivation		monobactam, carbapenem, cephalosporin
	yajC [1354543..1354881]	efflux		fluoroquinolone, cephalosporin, glycylcycline, penam, tetracycline, rifamycin, phenicol, triclosan
	mexL [1512834..1513472]	efflux		macrolide, tetracycline, triclosan
	mexJ [1513568..1514671]	efflux		macrolide, tetracycline, triclosan
	mexK [1514676..1517753]	efflux		macrolide, tetracycline, triclosan
	arnA [1649924..1651912]	target alteration		peptide
	mexP [1690136..1691293]	efflux		macrolide, carbapenem, tetracycline, acridine dye, diaminopyrimidine, phenicol, disinfecting

				agents and intercalating dyes
	mexQ [1691290..1694451]	efflux		macrolide, carbapenem, tetracycline, acridine dye, diaminopyrimidine, phenicol, disinfecting agents and intercalating dyes
	opmE [1694448..1695923]	efflux		macrolide, carbapenem, tetracycline, acridine dye, diaminopyrimidine, phenicol, disinfecting agents and intercalating dyes
	cpxR [2045209..2045886]	efflux		macrolide, fluoroquinolone, monobactam, aminoglycoside, carbapenem, cephalosporin, cephamycin, penam, tetracycline, peptide, aminocoumarin, diaminopyrimidine, sulfonamide, phenicol, penem
	cprS [2187670..2188965]	target alteration; efflux		peptide
	cprR [2188962..2189633]	target alteration; efflux		peptide
	adeF [2763881..2767057]	efflux	GI#8	fluoroquinolone, tetracycline
	muxA [2970528..2971808]	efflux		macrolide, monobactam, tetracycline, aminocoumarin
	muxB [2971805..2974936]	efflux		macrolide, monobactam, tetracycline, aminocoumarin

	muxC [2974933..2978043]	efflux		macrolide, monobactam, tetracycline, aminocoumarin
	opmB [2978040..2979539]	efflux		macrolide, monobactam, tetracycline, aminocoumarin
	oprN [3011209..3012627]	efflux		fluoroquinolone, diaminopyrimidine, phenicol
	mexF [3012624..3015812]	efflux		fluoroquinolone, diaminopyrimidine, phenicol
	mexE [3015834..3017078]	efflux		fluoroquinolone, diaminopyrimidine, phenicol
	soxR [3338679..3339149]	target alteration; efflux		fluoroquinolone, cephalosporin, glycylcycline, penam, tetracycline, acridine dye, rifamycin, phenicol, triclosan, disinfecting agents and intercalating dyes
	mexY [3743423..3746560]	efflux		macrolide, fluoroquinolone, aminoglycoside, carbapenem, cephalosporin, cephamycin, penam, tetracycline, acridine dye, phenicol, disinfecting agents and intercalating dyes
	parR [3996300..3997007]	efflux; reduced permeability to antibiotic	GI#12	macrolide, fluoroquinolone, monobactam, aminoglycoside, carbapenem, cephalosporin, cephamycin, penam,

				tetracycline, acridine dye, phenicol, penem, disinfecting agents and intercalating dyes
	parS [3997008..3998294]	efflux; reduced permeability to antibiotic	GI#12	macrolide, fluoroquinolone, monobactam, aminoglycoside, carbapenem, cephalosporin, cephamycin, penam, tetracycline, acridine dye, phenicol, penem, disinfecting agents and intercalating dyes
	mexN [4425285..4428395]	efflux		phenicol
	mexM [4428392..4429549]	efflux		phenicol
	pmpM [4559107..4560540]	efflux	GI#15	fluoroquinolone, aminoglycoside, benzalkonium chloride
	fosA [4811006..4811413]	inactivation		fosfomycin
	rsmA [5081427..5081612]	efflux		fluoroquinolone, diaminopyrimidine, phenicol
	crpP [5246731..5246928]	inactivation	CI#19	fluoroquinolone, ciprofloxacin
	oprJ [5386269..5387708]	efflux		macrolide, fluoroquinolone, aminoglycoside, cephalosporin, penam, tetracycline, aminocoumarin, diaminopyrimidine, phenicol
	mexD [5387714..5390845]	efflux		macrolide, fluoroquinolone, aminoglycoside,

				cephalosporin, penam, tetracycline, aminocoumarin, diaminopyrimidine, phenicol
	mexC [5390873..5391937]	efflux		macrolide, fluoroquinolone, aminoglycoside, cephalosporin, penam, tetracycline, aminocoumarin, diaminopyrimidine, phenicol
	catB7 [5786427..5787065]	inactivation		phenicol
	mexV [5954386..5955516]	efflux		macrolide, fluoroquinolone, tetracycline, acridine dye, phenicol, disinfecting agents and intercalating dyes
	mexW [5955567..5958623]	efflux		macrolide, fluoroquinolone, tetracycline, acridine dye, phenicol, disinfecting agents and intercalating dyes
	basS [6193592..6195025]	target alteration; efflux		peptide
	OpmH [6411491..6412939]	efflux		triclosan
	emrE [6433494..6433826]	efflux		aminoglycoside
	OXA-850 [7036044..7036832]	beta-lactamase		carbapenem, cephalosporin, penam
	QseB L71R [6192903..6193568]	mutation		peptide
	mexR [495984..496427]	target alteration; efflux		macrolide, fluoroquinolone, monobactam,

				carbapenem, cephalosporin, cephamycin, penam, tetracycline, peptide, aminocoumarin, diaminopyrimidine, sulfonamide, phenicol, penem
	nalC [1466771..1467412]	efflux		macrolide, fluoroquinolone, monobactam, carbapenem, cephalosporin, cephamycin, penam, tetracycline, peptide, aminocoumarin, diaminopyrimidine, sulfonamide, phenicol, penem
	mexT [3017309..3018223]	efflux		fluoroquinolone, diaminopyrimidine, phenicol
	mexS [3018444..3019463]	efflux		fluoroquinolone, diaminopyrimidine, phenicol
	mexZ [3741421..3742053]	efflux		macrolide, fluoroquinolone, aminoglycoside, carbapenem, cephalosporin, cephamycin, penam, tetracycline, acridine dye, phenicol, disinfecting agents and intercalating dyes
	nfxB [5392197..5392760]	efflux		macrolide, fluoroquinolone, cephalosporin, penam, tetracycline, aminocoumarin, diaminopyrimidine, phenicol
Streptococcus pneumonia PHRX1-2021				

PHRX1-2021: Chr	*pmrA* [1198848..1200047]	efflux		fluoroquinolone
	patB [1903612..1905378]	efflux		fluoroquinolone
	patA [1906169..1907863]	efflux		fluoroquinolone
	rlmA(II) [1934578..1935426]	target alteration		macrolide, lincosamide
